# A Case of Hemophilia

**Published:** 1906-01

**Authors:** R. F. Sheehan

**Affiliations:** Buffalo, N. Y.; Late House Physician at the Buffalo General Hospital


### A Case of Hemophilia.

By R. F. SHEEHAN, M. D., Buffalo, N. Y.

Late House Physician at the Buffalo General Hospital.

The patient A. M., age 16 years, entered the service of Dr. H. R. Hopkins, June 30, 1905, about 4 P. M. On June 26 he went to a dentist, who found that two molars complained of were too carious to be filled or crowned, and decided to extract them. They were drawn at 3 P. M. that day. The patient went home and soon began to bleed quite freely. He returned to the dentist, who packed the cavities; the bleeding, however, continued at intervals of from three to six hours until the present, patient loosing from 100 to 150 cc. of blood each time.

He had complained of severe headaches and great thirst, and was given all the fluid desired. At times he had become faint and stupid. Upon entrance June 30, at 4 P. M., the patient showed marked pallor, was very dull and extremely weak. Examination of the mouth revealed a blood clot, as large as an egg between the alveolar processes and projecting into the buccal cavity on the right side. When the clot and packing were removed, it showed that the second molar in the upper and lower jaws had been removed, and with the upper tooth a portion of the alveolar process.

There was considerable oozing from both cavities, especially the upper, because of the more lacerated gum and alveolar process. Pressure was made by means of small pads and bandages upon the common temporal, and upon the facial artery at the ramus. The mouth was then cleansed with swabs and a solution of hydrogen dioxide, the cavities repacked with cotton saturated with 1, 1000 solution adrenalin chloride.

It was necessary to repeat this every six to eight hours because of the clot formation which, however, was less each time. The presence of the clot caused a very unpleasant odor, which was most distressing to the patient, and prevented him from taking any nourishment by the mouth.

He was given small nutritive enemata every eight hours and between times an enema of normal salt solution 250 cc., in which was placed 1 gm. calcii chloridi. In addition, he had every 4 hours adrenalin 0.0006 hypodermically, for its effect in promoting blood coagulation. The effect upon the blood pressure being observed, it was upon entrance 108 mm. hg., and rose to 130 mm. hg. by 8 P. M. of the first day, at which point it was maintained. He was also given hypodermically, strychniae sulfatis 0.002, as a stimulant, every 4 hours.

July 1, at 8 P. M., the pressure was removed from the arteries. The next day, upon cleansing the mouth, the clot was found to be quite small, bleeding had ceased from the lower cavity, and there was but very little from the upper one. The latter was again packed as before, and the following day it was possible to leave out the packing. The mouth was kept clean by the liberal use of Dobell's solution and solution of hydogen dioxide. From this time the patient gradually improved.

He was given liquor arsenii et aurii bromidi et hydrargari iodidi (Park) 0.30 every 4 hours, which was increased 0.06 daily to 0.60., and tr. ferri chloridi 1. in a gelatine capsule three times daily. This was continued until July 20, when liq. ferri et ammonii acetatis 15., was substituted.

From July 7 he was given Vaughn's neuclein (hypodermicsol) 1. by mouth every 4 hours, which caused an increase in the number of leucocytes. After July 2, when mouth feeding was resumed, he had as much nourishment as he would take, especially milk, eggs and broths.

Upon dismissal July 30, the liq. ferri et ammonii acetatis was continued, and he was also given ovarian extract, to be taken for some time, as a prophylactic measure, following out a theory suggested by numerous observers (Vide Kinnicutt, *Medical Record*, June 10, 1905).

The blood examinations showed the following:

| | HEMOGLOBIN | ERYTHROCYTES | LEUCOCYTES |
|---|---|---|---|
| June 30 | 30% | 2,540,000 | 10,400 |
| July 4 | 15% | 1,900,000 | 5,400 |
| July 6 | 10% | 1,210,000 | 9,200 |
| July 10 | 20% | 1,500,000 | 9,000 |
| Jully 11 | 25% | 1,660,000 | 9,500 |
| July 13 | 30% | 2,320,000 | 9,600 |
| July 15 | 30-40% | 2,570,000 | 8,500 |
| July 17 | 40% | 2,730,000 | 7,800 |
| July 19 | 40-50% | 2,860,000 | 7,600 |
| July 21 | 40-50% | 2,980,000 | 7,200 |
| July 25 | 50% | 3,400,000 | 6,700 |

Stained specimen showed macrocytes, microcytes and poikilocytes, shadow corpuscles, with normoblasts, microblasts and a few megaloblasts, leucocytes:

| | |
|---|---|
| Polyneuclear.................... | 62% |
| Small lymphocytes.............. | 31% |
| Large lymphocytes.............. | 4% |
| Esinophiles.................... | 3% |
| | 100% |

The patient gives the following family history, which if reliable is somewhat contrary to the ordinary, in that the tendency is not confined mostly to the male issue.

In his brother, age 12 years, no tendency is exhibited. His mother, before 25 years, had frequent profuse epistaxsis. Maternal aunt, age 45, is a hemophiliac. He has had two uncles, one of whom was found dead in bed following epistaxsis; the other is also a hemophiliac. Two maternal cousins, boy and girl, both had the condition. A maternal aunt, who is free herself has a daughter with the tendency. His maternal grandmother bled very easily until after she was married. On the paternal side, there is no history of the condition.

Personal History: patient has never been sick. When two years old he shoved a pencil into his throat, from which injury he bled profusely, it being necessary to ligate the injured vessel. About a week ago bled considerably from slight cut on finger. He has never shown any signs of joint involvement.

479 Delaware Avenue.

